# Transcriptome analysis reveals liver metabolism programming in kids from nutritional restricted goats during mid-gestation

**DOI:** 10.7717/peerj.10593

**Published:** 2021-01-29

**Authors:** Chao Yang, Xiaoling Zhou, Hong Yang, Kefyalew Gebeyew, Qiongxian Yan, Chuanshe Zhou, Zhixiong He, Zhiliang Tan

**Affiliations:** 1 CAS Key Laboratory for Agro-Ecological Processes in Subtropical Region, National Engineering Laboratory for Pollution Control and Waste Utilization in Livestock and Poultry Production, South-Central Experimental Station of Animal Nutrition and Feed Science, Institute of Subtropical Agriculture, The Chinese Academy of Sciences, Changsha, Hunan, China; 2 University of Chinese Academy of Sciences, Beijing, China; 3 College of Animal Science, Tarim University, Alaer, Xinjiang, China; 4 Hunan Co-Innovation Center of Animal Production Safety, Changsha, Hunan, China

**Keywords:** Goat, Maternal undernutrition, Hepatic metabolism, RNA-sequencing

## Abstract

**Background:**

Maternal nutrient restriction during pregnancy causes a metabolic disorder that threatens the offspring’s health in humans and animals. However, the molecular mechanism of how undernutrition affecting hepatic metabolism of fetal or postnatal offspring is still unclear. We aimed to investigate transcriptomic changes of fetal livers in response to maternal malnutrition in goats during mid-gestation and to explore whether these changes would disappear when the nutrition was recovered to normal level during mid-gestation using goats (*Capra hircus*) as the experimental animals.

**Methods:**

Fifty-three pregnant goats were subjected to a control (100% of the maintenance requirements, CON) or a restricted (60% of the maintenance requirements on day 45 to day 100 of gestation and then realimentation, RES) diet. A total of 16 liver samples were collected from fetal goats on day 100 of gestation and goat kids of postnatal day 90 to obtain hepatic transcriptional profiles using RNA-Seq.

**Results:**

Principal component analysis of the hepatic transcriptomes presented a clear separation by growth phase (fetus and kid) rather than treatment. Maternal undernutrition up-regulated 86 genes and down-regulated 76 genes in the fetal liver of the FR group as compared to the FC group. KEGG pathway analysis showed the DEGs mainly enriched in protein digestion and absorption, steroid biosynthesis, carbohydrate digestion and absorption and bile secretion. A total of 118 significant DEGs (fold change > 1.2 and FDR < 0.1) within KR vs. KC comparison was identified with 79 up-regulated genes and down-regulated 39 genes, and these DEGs mainly enriched in the biosynthesis of amino acids, citrate cycle, valine, leucine and isoleucine biosynthesis and carbon metabolism.

**Conclusion:**

Hepatic transcriptome analysis showed that maternal undernutrition promoted protein digestion and absorption in the fetal livers, while which restrained carbohydrate metabolism and citric acid cycle in the livers of kid goats after realimentation. The results indicate that maternal undernutrition during mid-gestation causes hepatic metabolism programming in kid goats on a molecular level.

## Introduction

Maternal and child malnutrition is a common public health problem in underdeveloped regions and countries, leading to severe disease burden and higher mortality (Organization, 2008). Inadequate nutrients intake in gestation restricts fetal growth in the utero, reduces birth weight and increases the risk of metabolic disorders for the offspring (Barker, 1997; Odle et al., 2017). With the Developmental Origins of Health and Disease hypothesis emerging in the early 1990s, it was proven that causal links exist between prenatal nutrition and metabolic diseases after birth, such as type 2 diabetes, obesity and cardiovascular diseases (Hales & Barker, 1992; Popkin, Adair & Ng, 2012). Meanwhile, it has been verified that maternal undernutrition has long-lasting effects on subsequent growth and metabolism, which insults the function of organs or tissues in offspring; this process is called “nutritional programming” (McMillen & Robinson, 2005).

Liver is a crucial metabolic organ in animals, which senses and processes the maternal nutrients in growing fetus (Wesolowski & Jr, 2016). During midgestation, the principal function of liver becomes to hepatogenesis with a geometric growth of hepatoblast proliferation which mainly affected by nutritional level (Ober & Lemaigre, 2018). Sufficient nutrients intake for intrauterine fetus can ensure normal development of hepatic function and hinder the metabolic disorders at the juvenile stage. However, maternal nutrient deficiencies disturb gene expression which involved in hepatic energy homeostasis and finally increase the risk of metabolic syndrome in later life (Canani et al., 2011; Godfrey, Inskip & Hanson, 2011; Sebert et al., 2011). Besides, liver also plays a vital role in immunity because blood containing substantial antigens from the gastrointestinal tracts circulates through the vascular system in the liver that has a contribution in building an immune barrier to defense pathogen (Racanelli & Rehermann, 2004; Stamataki & Swadling, 2020). It has been stressed that adequate maternal nutrition can pledge the normal development of human immunity during gestation or after birth (Chandra, 2002).

Several studies have been extensively investigated the consequences of peri-conceptional undernutrition in rats (Franko, Forhead & Fowden, 2009), cattle (Reynolds et al., 2010) and sheep (Heasman et al., 1999). The results have shown that maternal nutrient restriction in the animals mentioned above led to metabolic disturbances, which results in the programming of genetic and phenotypic changes on their offspring. Intrauterine nutritional restriction (50% of total digestible nutrients) from day 28 to day 78 of gestation decreases blood glucose concentration and body size of fetuses, while the right and left-ventricle and liver weights of restricted fetuses elevates (Vonnahme et al., 2003). In addition, Sen (2015) reported that maternal nutrition restriction of ewes during mid-gestation reduces the postnatal growth and muscle fiber development of their offspring. It is possible that intrauterine malnutrition affects the nutrients involving in the bones, muscles and lipid genesis of fetuses when fetal organogenesis, placental growth and vascularization display a rapid development during early- or mid-gestation (Reynolds & Redmer, 1995). Meanwhile, previous researches in our team have noted that nutritional restriction during midgestation changes the hepatic metabolic pathways and immune processes in the fetal goats; however, this effect disappears in the kid goats after the nutritional level recovered to a normal level (Chen et al., 2019; Zhou et al., 2019). Additionally, energy or protein restriction (40% of the maintenance requirements) during late gestation reduces the immune capacity in neonatal goats and kid goats (He et al., 2009).

Despite maternal nutrients restriction disordering the hepatic metabolism has been extensively investigated in humans and animals (Hardy, 2017), the genomic mechanisms through which nutrients deficiency complicates hepatic metabolic disorders remain unclear. With the advent of transcriptome, high-throughput RNA sequencing (RNA-Seq) has been widely used to identify critical genes and pathways related to physiological processes. However, knowledge on the effect of maternal nutrients restriction during midgestation on the hepatic transcriptome profile of fetal and kid goats has not been reported. Using goats (*Capra hircus*) as the experimental animals, our first objective was to reveal the effects of maternal nutrients restriction on the hepatic function of fetal goats, and the second was to investigate whether maternal nutrients restriction causes metabolic programming of kid goats using RNA-seq.

## Materials and Methods

### Animal ethics

All procedures for the animal trial were in accordance with the Guide for the Animal Care and Use and also approved by the Animal Care Committee of Institute of Subtropical Agriculture, Chinese Academy of Sciences, Changsha, China (No. KYNEAAM-2015-0009). All goats used in this study belonged to local farm (Mulei Black Goats Breeding Farm) in Liuyang, Changsha, China, and the owner (Mr. Yuanwei Yang) of the farm agreed to our experiment.

### Animal, treatments and sample collection

Fifty-three pregnant Xiangdong black goats (based on the homogeneity of their pregnancy duration 45 ± 3 d and their parity) were selected and randomly allocated into the control group (CON, 100% of the maintenance requirements, *n* = 25) and nutritional restriction group (RES, 60% of the maintenance requirements, *n* = 28). The maintenance requirements were the recommended values according to the Feeding Standard of Meat-producing Sheep and Goats of China (2004). The ingredients and nutrient composition of the experimental diet on a dry matter basis are shown in Table 1. Goats were penned individually in stainless cages for 14 days to adapt the diet and environment and fed twice per day at 08:00 and 16:00 with a 50:50 concentrate-to-forage ratio, and had free access to water. Dams in the RES group were provided 60% of the feed allowance of the CON group from d 45 to 100 of gestation, and the actual feed allowance of the CON and RES groups for each dam were 1.04 and 0.62 kg/d, respectively. Dams were examined using portable ultrasonography (Aloka SSD-500 with a 5-MHz linear probe Aloka, Shanghai, China) to determine the litter size at d 100 of gestation, except four dams aborted from each group. Pregnant dams with similar initial BW and equal litter size (singleton pregnancy) were selected from each group and slaughtered by a registered veterinarian with an intravenous injection of sodium pentobarbital (50 mg/kg BW) before feeding in the morning. Five and four fetuses from the CON group and the RES group were collected immediately, and liver samples were then removed and labeled as FC and FR, respectively. All fetal liver samples were washed three times with phosphate-buffered saline (pH 7.4; Solarbio LIFE SCIENCES, Beijing, China), and stored in liquid nitrogen.

**Table 1:** Ingredients and nutrient composition of experimental diet for pregnant goats.

**Items**	**Content**
Ingredients (%)	
Fresh-mowed *Miscanthus*	50.00
Maize	33.50
Soybean meal	10.33
Fat power	4.00
Calcium carbonate	0.49
Calcium bicarbonate	0.46
Sodium chloride	0.22
Premix^a^	1.00
Nutrient composition^b^, % of DM basis	
Metabolic energy (MJ/kg)	11.78
Crude protein	12.05
Ether extract	8.97
Neutral detergent fiber	64.44
Acid detergent fiber	28.32
Ash	5.89
Calcium	0.53
Phosphorus	0.20

**Notes.**

a Premix was provided per kilogram of total diet DM, and the composition was as follows: 95,000 IU of vitamin A, 17,500 IU of vitamin D, 18,000 IU of vitamin E, 119 g of MgSO_4_.H_2_O, 2.5 g of FeSO_4_.7H_2_O, 0.8 g of CuSO_4_. 5H_2_O, 3 g of MnSO_4_.H_2_O, 5 g of ZnSO_4_. H_2_O, 10 mg of Na_2_.SeO_3_, 40 mg of KI, 30 mg of CoCl_2_.6H_2_O.

b Metabolic energy was calculated, and the others were measured values.

The nutritional level for dams of the RES group recovered to 100% of the maintenance requirements from d 101 of gestation and the management was described above during the following experimental period. After parturition, neonatal singleton goats were lived with and nursed by their dams and were gradually weaned between d 50 and 60. Kids were separated from dams on daytime (08:00 to 16:00) and were supplemented with a mixed diet, including 80% starter and 20% fresh *Miscanthus* spp., and had free access to water. Kids from the CON and RES group were labeled as KC and KR and moved onto a big pen following weaning on day 60. All kid goats were provided the diet (80% starter and 20% fresh *Miscanthus spp.*) twice a day at 08:00 and 16:00. The ingredients and nutrient composition of the kid’s diet on a dry matter basis are shown in Table S1. Between d 61 to d 90, the average daily feed intake (on a dry matter basis) of kids in the CON and RES group was 0.31 and 0.29 kg/d, respectively. Three and four eligible singleton kids from CON and RES group were slaughtered at d 90 after birth, respectively, and the procedures of slaughter and liver sample collection were the same to above description.

### RNA extraction

Approximate 100 mg frozen liver tissues were ground to fine powder in liquid nitrogen using a frozen mortar and transferred into a 1.5 mL DNA/RNase free tube, then added 1 mL Trizol reagent (Invitrogen, CA, USA) for total RNA extraction following the manufacturer’s instruction. The purity and concentration of total RNA were determined using the NanoPhotometer^®^ spectrophotometer (IMPLEN, CA, USA) and the Qubit^®^ 2.0 Fluorometer (Life Technologies, CA, USA), respectively. Additionally, the Bioanalyzer 2100 system was used to check the integrity of the RNA with an RNA 6000 Nano LabChip Kit (Agilent Technologies, CA, USA). RNA samples, which have an integrity number (RIN) greater than 7.0 (and the ratio of 28S/18S ranging from 1.8 to 2.2), were used for library construction.

### RNA library construction and sequencing

Extracted total RNA (2 µg) for each sample was subjected to isolate Poly (A) mRNA with poly-T oligo attached magnetic beads (Invitrogen, CA, USA) for purification. The mRNA was fragmented into small pieces using divalent cations under elevated temperature. Then the RNA fragments were reverse-transcribed to create the final cDNA library following the protocol of the mRNA Seq Sample Preparation Kit (Illumina, San Diego, USA). Following reverse transcription, the cDNA was subjected to 5′-end repair and 3′-end adenylation, then conducted PCR to obtain the paired-end libraries (250–270 bp). cDNA libraries were verified and quantified using an Agilent 2200 TapeStation (Agilent Technologies, CA, USA) and a Qubit^®^ 2.0 Fluorometer (Life Technologies, CA, USA), respectively. Finally, the individual libraries were sequenced on the Illumina HiSeq 4000 platform (Illumina) to obtain 100 bp paired-end reads.

### RNA sequencing data processing and normalization

Raw data were evaluated by FAST-QC software (version 0.10.0) to conduct quality control testing (Santos et al., 2018b). Subsequently, the HISAT2 package was used to map raw data to goat genome reference (ARS1) with default parameters (Bickhart et al., 2017; Daehwan, Ben & Salzberg, 2015). The mapped reads of each sample were assembled using StringTie; all transcripts from one sample were merged to reconstruct a comprehensive transcriptome (Mihaela et al., 2015). StringTie and Ballgown were used to estimate the expression level of each gene by calculating FPKM (fragments per kilobase of transcript per million fragments reads) based on the length of the gene and the number of reads mapped to the gene (Frazee et al., 2015). Principal Component Analysis (PCA) between fetus group and kid group was conducted with cluster samples based on gene expression data using R (R version 3.4.2).

### Identification of differential expression and functional enrichment analyses

We performed differential expression analysis to investigate the effects of nutritional levels in she-goats during mid-gestation on hepatic transcriptome profiles of their fetuses and kids using the Bioconductor package DESeq2 (v 1.22.2, *Love, Huber & Anders, 2014)*. Two contrasts including FC vs. FR and KC vs. KR were performed to identify the significant differentially expressed genes (DEGs), which were estimated with —foldchange—>1.2 and FDR <0.1 as described by *He et al. (2018)*. Gene ontology (GO) enrichment analysis was performed to classify the function of the top 3000 highly expressed genes and DEGs in categories including molecular function, biological process and cellular component using the GOseq R package. Significant GO terms were declared at the *P*-value <0.05 (Young et al., 2010). Furthermore, DEGs and top 3000 highly expressed genes enrichment in the Kyoto Encyclopedia of Genes and Genomes (KEGG) pathways were estimated by KOBAS software and significant KEGG pathways were selected at *P*-value <0.05 (Mao, Tao & Wei, 2005).

### Analysis of key genes associated with hepatic metabolism

To estimate whether maternal malnutrition in goats during mid-gestation affected hepatic metabolism in fetuses and whether this effect continued to disorder kids’ hepatic metabolism when nutritional level recovered, the sequencing data of genes involved in hepatic metabolism was further analyzed. Genes involved in gluconeogenesis, lipid metabolism and cellular energy metabolism were selected according to functional annotation (Keogh et al., 2016; Wood et al., 2013) and heatmap was drawn with log2 FPKM of expression of genes using R (R version 3.4.2).

### Real-time quantitative PCR validation of RNA-seq data

The real-time quantitative PCR (qPCR) validation of the RNA-seq results was performed to determine the expression of genes involved in the metabolic process and immune response in the liver of fetal and kid goats. The total RNA extraction protocol of the same animals used in the current study was similar to what was described above. The eligible RNA was used to synthesize cDNA using a commercial kit (AG11705, Accurate Biology, Changsha, China) according to the manufacture’s specification. The PCR premix was 10 µL reaction system, containing 5 µL 2X SYBR^®^ Green *Pro Taq* HS Premix (Accurate Biology, Changsha, China), 0.2 µL each primer, 1.0 µL cDNA template and 3.6 µL RNase free water. The reaction was conducted in a fluorescence LightCycler 480 II (Roche, Basel, Switzerland) with the following program: 95 °C for 30 s, followed by 40 cycles of 95 °C for 5.00 s, annealing at 60 °C for 30 s. After the amplification process, a melting curve was generated with an increasing temperature of 0.50 °C every 5.00 s starting at 65 °C to 95 °C. The expression levels of each candidate genes were normalized to *β*-actin, and the relative expression of each gene was calculated by the 2^−ΔΔ*Ct*^ method. The data of gene expression of fetal or kid liver were analyzed using the independent-sample *t* test, and *P* <  0.05 were considered statistically significant. The primer sequences for hepatic metabolism and immunity related DEGs were deposited in Table S2.

## Results

### Hepatic transcriptome profiles of fetuses and kids

**Figure 1:**
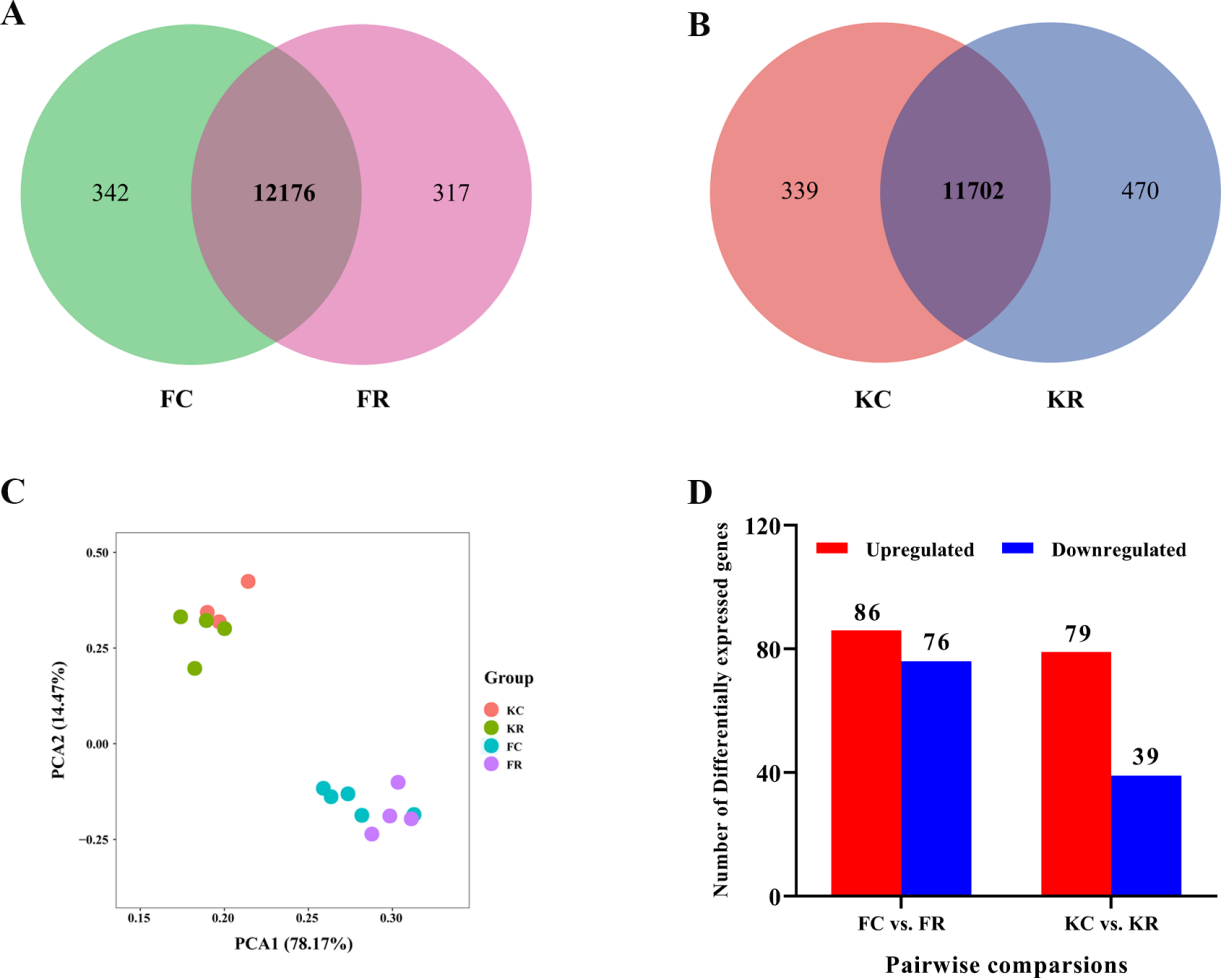
Hepatic transcriptomic profiles of expressed genes (with average FPKM > 1 of the group and FPKM > 1 in at least one animal) in goat fetuses and kids. (A) Venn diagram showing the overlap of expressed genes in livers of goats from FC and FR groups. (B) Venn diagram showing the overlap of expressed genes in livers of goats from KC and KR groups. (C) PCA plot of expressed genes for the liver samples of goats from FC, FR, KC and KR groups. The *X* and *Y*-axis represent the first two principle components. The percentage value in the bracket represents the percentage of variance explained by that principle component. (D) Number of differential expressed genes between FC and FR group and KC and KR group.

To investigate the hepatic transcriptome response to maternal nutritional restriction during mid-gestation in fetal and kid goats, we obtained a total of 733.9 million valid reads from all 16 samples, with an average of 45.9 ±  1.17 million reads per sample (File S3) based on the Illumina HiSeq 4000 platform. As a result, approximately 43.53, 45.54, 44.38 and 50.23 million high-quality 100-bp paired-end reads were obtained from goats of FC, FR, KC and KR group, respectively. For above each group, 96.65%, 96.58%, 96.82% and 96.23% of reads could be mapped to the goat reference genome, 65.14%, 63.75%, 65.86% and 63.85% of these mapped reads could be aligned with unique genes explicitly (Table 2). Based on the normalized data, 12,518, 12,493, 12,041 and 12,172 expressed genes (with average FPKM >1 of the group and FPKM >1 in at least one animal) were detected in the livers of FC, FR, KC and KR group, respectively. Among these genes, 12,176 genes were commonly expressed in FC and FR groups, 342 and 317 genes were solely identified in FC and FR goats (File S4, Fig. 1A), respectively; meanwhile, a total of 11,702 genes were collectively expressed in KC and KR groups, in which 339 and 170 genes were uniquely expressed in KC and KR group, respectively (File S4, Fig. 1B). The principal component analysis of the transcriptome profiles didn’t present a clear separation in fetal goats by treatments, which was the same as that in kid goats (Fig. 1C). The most significant GO term of the top 3,000 highly expressed genes was oxidation–reduction process. The top 3,000 highly expressed genes mainly enriched in metabolism-related pathways such as oxidative phosphorylation, carbon metabolism, fatty acid degradation, valine, leucine and isoleucine degradation and citrate cycle (File S5).

**Table 2:** Statistics of the sequencing reads alignment to the reference genome.

**Statistics term**	**FC**	**FR**	**KC**	**KR**
Valid reads	43529259	45542586	48932146	50231888
Mapped reads	42070428	43983092	47403576	48356456
Mapping rate (%)	96.65	96.58	96.87	96.23
Unique mapped reads	28366328	28929147	29058255	32268886
Unique mapping rate (%)	65.14	63.75	60.80	65.55
Unmapped reads	1458831	1559494	1528570	1875432
Multi mapped reads	13704099	15053945	18345322	16087570

### Effect of maternal malnutrition in goats during mid-gestation on hepatic transcriptome in fetal goats

**Figure 2:**
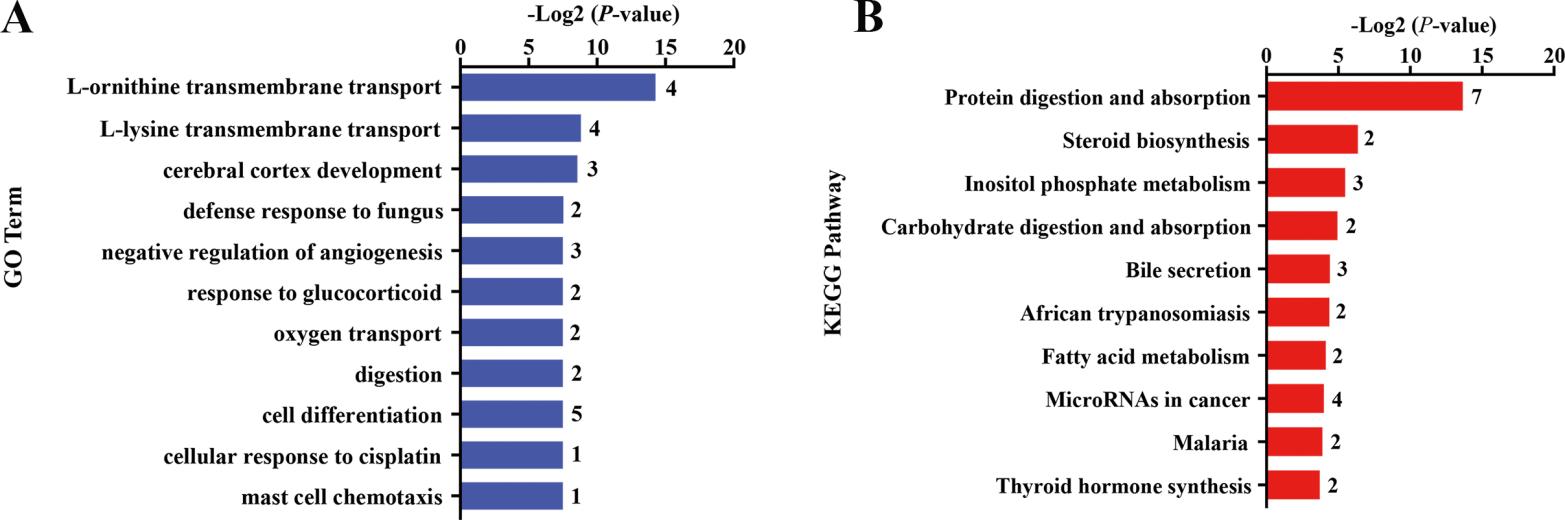
Gene ontology (GO) and KEGG pathway analyses of the DEGs in the liver of fetal goats. (A) The significant GO terms (biological processes) associated with the identified DEGs in fetal goats between FC and FR groups. The vertical axis represents the GO category, and the horizontal axis represents the -Log2 (*P*-value) of the significant GO terms. The number represented amount of DEGs enriched in the corresponding GO terms. (B) The significant pathways associated with the identified DEGs in fetal goats between FC and FR groups. The vertical axis represents the pathway category, and the horizontal axis represents the -Log2 (*P*-value) of the significant pathways. The number represented amount of DEGs enriched in the corresponding pathways.

Differential gene expression analysis was performed between FC and FR group to evaluate the hepatic transcriptome changes in fetuses that had undergone maternal malnutrition in goats during mid-gestation. A total of 162 significant DEGs (—foldchange—>1.2 and FDR <0.1) were detected in the fetal livers for FC vs. FR. In detail, 76 genes showed higher expression in the FC than the FR group, whereas 86 genes exhibited higher expression in FR than the FC group (Fig. 1D, File S6). To obtain biological functions of the above DEGs, GO and KEGG pathway analyses were conducted by querying each DEG identified in the fetal livers. The results showed that the significant GO terms of DEGs in the biological process were L-ornithine transmembrane transport, L-lysine transmembrane transport, cerebral cortex development, defense response to fungus, native regulation of angiogenesis, response to glucocorticoid, oxygen transport, digestion and cell differentiation (*P* <  0.05, Fig. 2A). The upregulated DEGs between the FC and FR groups were mainly involved in cell differentiation, cerebral cortex development, brain development, digestion, negative regulation of receptor signaling pathway via JAK-STAT, neuron differentiation, negative regulation of neuron apoptotic process and chemical synaptic transmission (at least two DEGs, *P* <  0.05, File S7). However, the downregulated DEGs were mainly associated with L-ornithine transmembrane transport, L-lysine transmembrane transport, negative regulation of angiogenesis, defense response to fungus, platelet activation, negative regulation of phosphatase activity and cellular response to hypoxia (at least two DEGs, *P* <  0.05, File S7).

Pathway analysis showed that the significant KEGG pathways were protein digestion and absorption, steroid biosynthesis, inositol phosphate metabolism, carbohydrate digestion and absorption and bile secretion (*P* <  0.05, Fig. 2B). Upregulated DEGs between the FC and FR groups were mainly involved in protein digestion and absorption, bile secretion, steroid biosynthesis, carbohydrate digestion and absorption, thyroid hormone synthesis, ABC transporters and insulin secretion (at least two DEGs, *P* <  0.05, File S8), while downregulated DEGs were mainly enriched in protein digestion and absorption, MicroRNAs in cancer, cytokine-cytokine receptor interaction, inositol phosphate metabolism and NF-kappa B signaling pathway (at least two DEGs, *P* <  0.05, File S8).

### Effect of maternal undernutrition in goats during mid-gestation on hepatic transcriptome in kid goats

**Figure 3:**
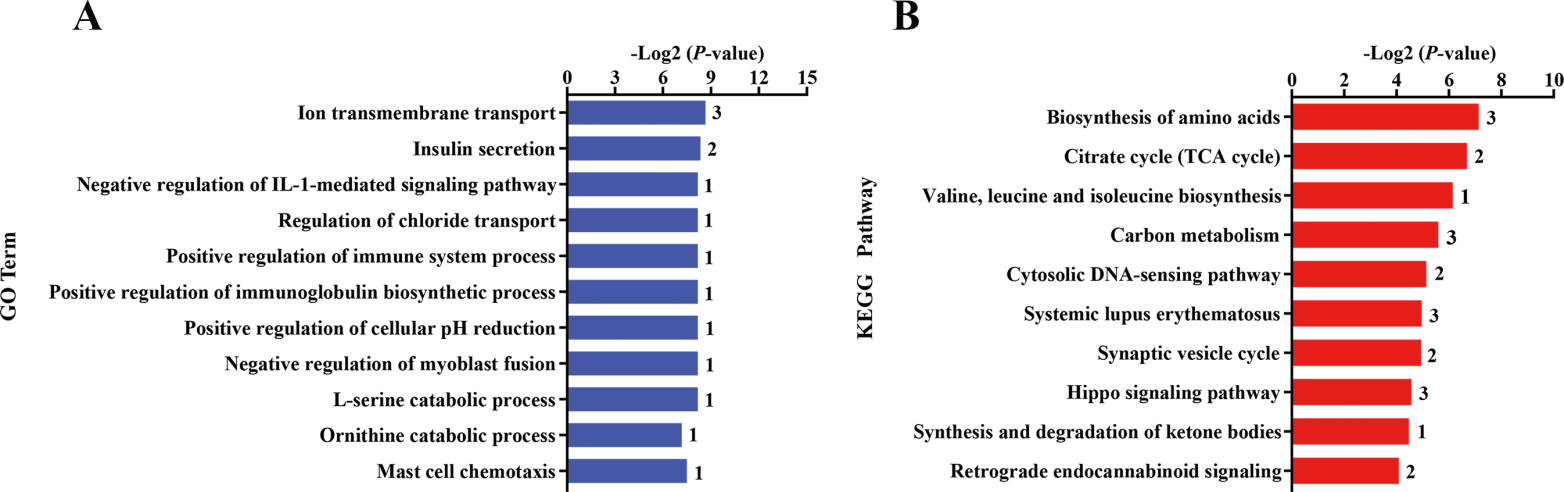
Gene ontology (GO) and KEGG pathway analyses of the DEGs in the liver of kid goats. (A) The significant GO terms (biological processes) associated with the identified DEGs in kid goats between KC and KR groups. The vertical axis represents the GO category, and the horizontal axis represents the -Log2 (*P*-value) of the significant GO terms. The number represented amount of DEGs enriched in the corresponding GO terms. (B) The significant pathways associated with the identified DEGs in kid goats between KC and KR groups. The vertical axis represents the pathway category, and the horizontal axis represents the -Log2 (*P*-value) of the significant pathways. The number represented amount of DEGs enriched in the corresponding pathways.

The nutritional level recovered at d 101 of gestation, and kids in the KR group received the same diet and management as that in the KC group. After differential expressed gene analyses, we identified 118 significant DEGs (—foldchange—>1.2 and FDR <0.1) within KC vs. KR group. Of these DEGs, the expression of 79 genes was upregulated, while the expression of 39 genes was downregulated in the KR group when compared to the KC group (Fig. 1D, File S6). Functional classification showed that DEGs of the above comparison mainly enriched in the GO terms of the biological process were ion transmembrane transport, insulin secretion, negative regulation of IL-1-mediated signaling pathway, regulation of chloride transport, positive regulation of immune system process, positive regulation of immunoglobulin biosynthetic process and so on (*P* <  0.05, Fig. 3A). Upregulated DEGs were mainly enriched in signal transduction, insulin secretion, L-ornithine transmembrane transport, L-lysine transmembrane transport, regulation of cell population proliferation and intracellular protein transport; however, downregulated DEGs were significantly enriched in chloride transmembrane transport and ion transmembrane transport (at least two DEGs, *P* <  0.05, File S9).

Pathway analysis showed that DEGs between the KC and KR groups were biosynthesis of amino acids, citrate cycle, valine, leucine and isoleucine biosynthesis, carbon metabolism, cytosolic DNA-sensing pathway, systemic lupus erythematosus, synaptic vesicle cycle, hippo signaling pathway and synthesis and degradation of ketone bodies (*P* <  0.05, Fig. 3B). In details, upregulated DEGs were mostly involved in MicroRNAs in cancer, cytokine-cytokine receptor interaction, PI3K-Akt signaling pathway, cytosolic DNA-sensing pathway, retrograde endocannabinoid signaling, Toll-like receptor signaling pathway and Hippo signaling pathway (at least two DEGs, *P* <  0.05, File S10), while downregulated DEGs were principally enriched in citrate cycle, biosynthesis of amino acids, gap junction, carbon metabolism and complement and coagulation cascades (at least two DEGs, *P* <  0.05, File S10).

### Expression of key genes associated with hepatic metabolism in response to nutritional restriction of fetal and kid goats in early life

**Figure 4:**
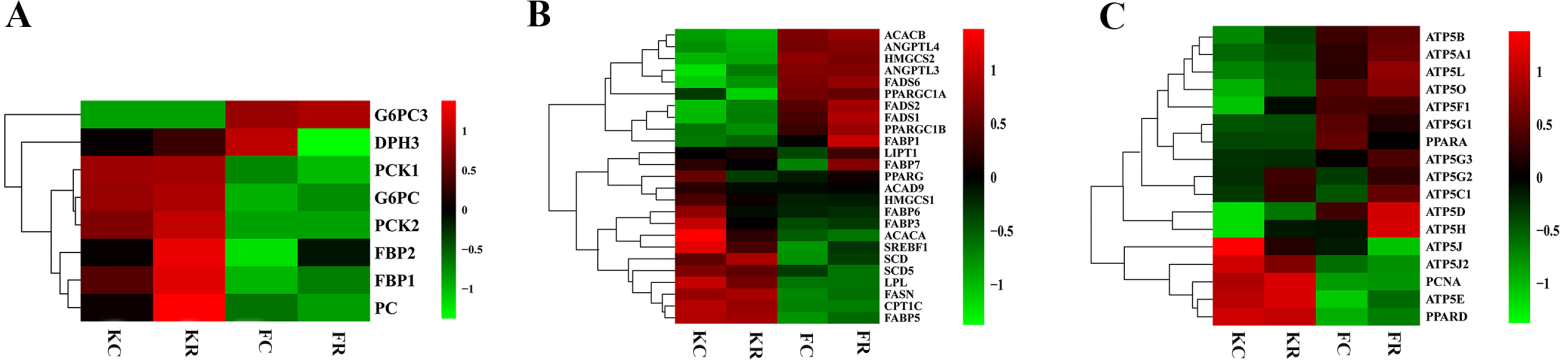
Expression (log2 FPKM) of genes involved in hepatic metabolism of goats from each group. (A) Heat map for the expression of genes involved in carbohydrate metabolism of goats from each group. (B) Heat map for the expression of genes involved in lipid metabolism of goats from each group. (C) Heat map for the expression of genes involved in cellular energy metabolism of goats from each group. Green color represents lower expression and red color represents higher expression. Data are expressed as means ±SD, *P < 0.05.

**Figure 5:**
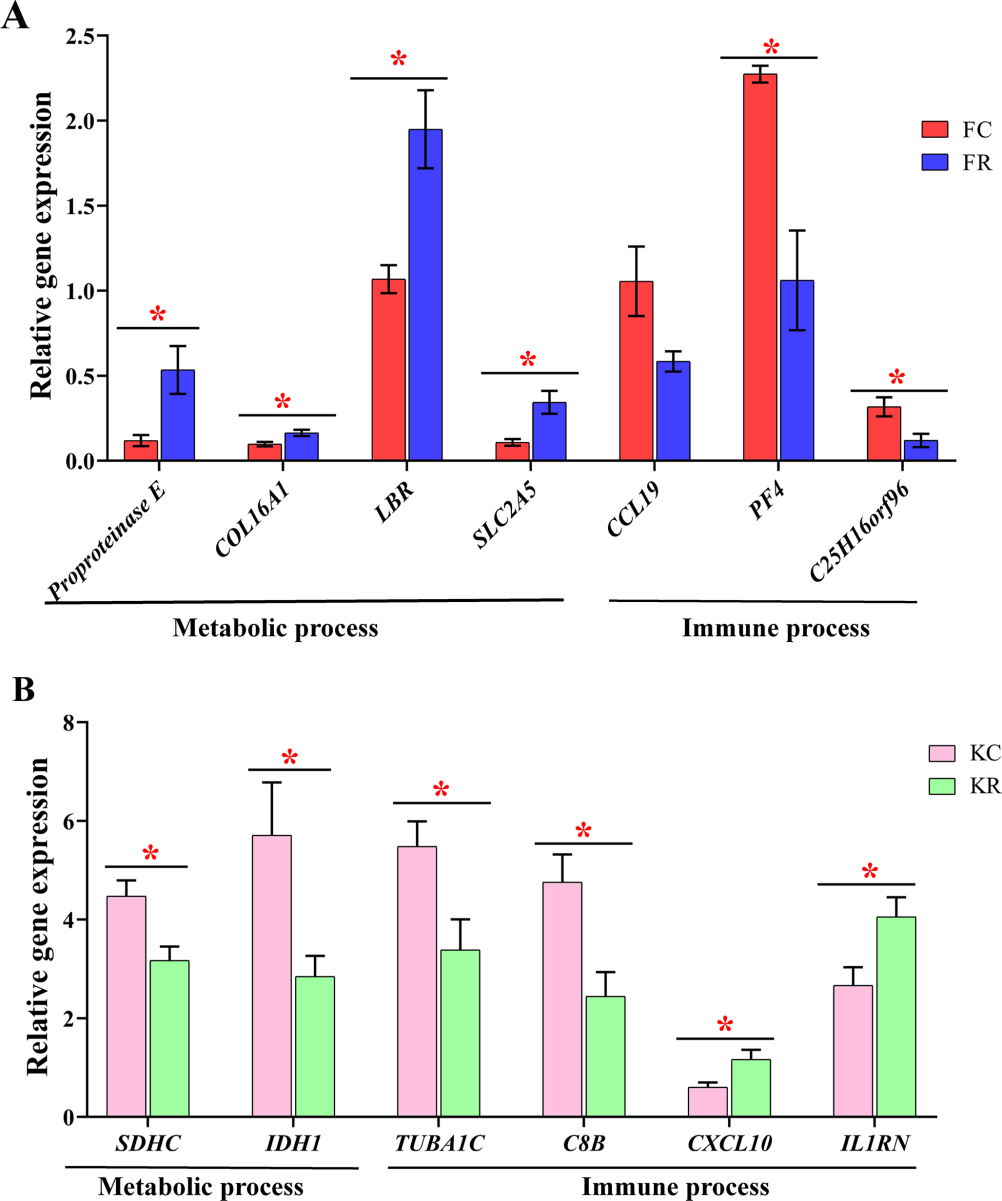
Validation of metabolic and immune DEGs in the liver of fetal and kid goats by quantitative real-time PCR (qRT-PCR). (A) The relative gene expression of DEGs related to hepatic metabolism and immunity by qRT-PCR in the fetal goats. (B) The relative gene expression of DEGs related to hepatic metabolism and immunity by qRT-PCR in the kid goats. Values are presented as mean with their standard errors. *P < 0.05.

To evaluate the effect of nutritional restriction during mid-gestation on hepatic metabolism of fetal goats and kid goats, we performed a further analysis using RNA-sequencing data and plotted the heatmap using average FPKM values of the genes, which involved in gluconeogenesis, lipid metabolism and cellular energy metabolism, respectively (Fig. 4, File S11). However, the expression of the above genes was not affected by nutritional restriction (FDR >0.1). The results of pathway analyses showed that 13 upregulated DEGs (*LOC102186539*, *LOC102188098*, *COL16A1*, *HRCT1*, *LOC108636568*, *RUNDC3A*, *LOC102173859*, *LBR*, *LIPN*, *LOC102178379*, *LRP2*, *RUNDC3A*, *SNAP25* and *ACSL4*) mainly enriched in protein digestion and absorption, bile secretion, steroid biosynthesis, carbohydrate digestion and absorption, thyroid hormone synthesis, ABC transporters, insulin secretion and fatty acid biosynthesis as well as 3 downregulated DEGs (*TMEM19*, *PEG3* and *SHISA9*) involved in protein digestion and absorption in the liver of fetal goats (Table 3). However, 3 downregulated DEGs (*SDHC*, *IDH1* and *OTC*) enriched in citrate cycle, biosynthesis of amino acids, carbon metabolism, 2-Oxocarboxylic acid metabolism and arginine biosynthesis in the liver of kid goats (Table 3). To validate the expression of DEGs related to hepatic metabolism and immunity, qPCR was performed using corresponding RNA samples in the fetal and kid goats. The results showed that the expression of *Proproteinase E, COL16A1, LBR* and *SLC2A5* were significantly upregulated by maternal undernutrition in the liver of fetal goats (*P* <  0.05, Fig. 5A), however, the mRNA level of *CCL19*, *PF4* and *C25H16orf96* were downregulated in the liver of fetal goats in KR group (*P* <  0.05, Fig. 5A). In addition, maternal undernutrition downregulated the expression of *SDHC*, *IDH1*, *TUBA1C* and *C8B* (*P* <  0.05, Fig. 5B), while the mRNA level of *CXCL10* and *IL1RN* were significantly upregulated in the liver of kid goats in KR group (*P* <  0.05, Fig. 5B). The results of qPCR analyses for hepatic metabolism and immune related DEGs are similar to that obtained from transcriptomic analyses.

**Table 3:** Expression statistics (average FPKM) of the representative metabolism related DEGs in the of liver fetal and kid goats.

**Gene name**	**Gene expression**		**Fold change (log2)**	***P*value**	**FDR**
	**CON**	**RES**			
Fetal goats					
*LOC102186539*	14.45	22.12	0.614	<0.001	0.034
*LOC102188098*	39.79	48.27	0.279	<0.001	0.025
*COL16A1*	11.11	23.25	1.065	0.001	0.057
*HRCT1*	10.37	12.52	0.271	<0.001	0.037
*RUNDC3A*	2.22	3.16	0.510	0.001	0.051
*LOC102173859*	4.92	9.57	0.959	<0.001	0.030
*LBR*	18.45	22.23	0.269	0.002	0.092
*LIPN*	0.18	0.71	1.954	<0.001	0.033
*SLC2A5*	9.88	15.66	0.664	<0.001	0.034
*TMEM19*	0.12	0.09	−0.376	<0.001	0.044
*PEG3*	150.63	123.04	−0.292	0.001	0.056
*SHISA9*	1.58	0.89	−0.824	0.001	0.064
Kid goats					
*SDHC*	46.84	36.68	−0.353	<0.001	0.093
*IDH1*	39.84	27.99	−0.510	<0.001	0.051
*OTC*	60.38	38.96	−0.636	0.001	0.065

## Discussion

This is the first study to describe the characteristics of hepatic transcriptome profiles as well as the successive effects of maternal nutrients restriction during mid-gestation on the hepatic transcriptome profiles of goat fetuses and juveniles. To our knowledge, early- and mid-gestation are crucial phases to humans or other mammals because the fetus displays a rapid development of organ and tissue as well as corresponding functions, so adequate nutrients intake during this period ensures good fetal growth and decreases the risk of metabolic diseases (Ford, 1995; Reynolds & Redmer, 1995). We analyzed the transcriptome profiles of goat fetuses and juveniles that undergone maternal nutrients restriction during mid-gestation to investigate whether malnutrition affected the hepatic metabolism of nutrients, and evaluated whether maternal undernutrition resulted in hepatic metabolic programming of juveniles.

### Hepatic transcriptome profiles in fetal and kid goats

As regard to hepatic transcriptome profiles of fetuses and kids, a total of 12,518, 12,493, 12,041 and 12,172 core genes were detected in the livers of FC, FR, KC and KR group, which is similar to the descriptions of human, mouse and cattle liver tissue by RNA-seq (Mccabe et al., 2012; Ramsköld et al., 2009). Studies have noted that KEGG enriched pathways for the hepatic transcriptional profile in cattle were metabolic pathways, ribosome, steroid hormone biosynthesis and other amino acids metabolic pathways (Keogh et al., 2016). It supported the present findings that the top 3,000 highly expression genes mainly enriched in pathways including oxidative phosphorylation, carbon metabolism, fatty acid degradation, valine, leucine and isoleucine degradation and citrate cycle. These results suggested that goats experience rapid development of organ, tissue and the corresponding function during early life stage. In these stages, goats consume more energy and proteins in which amino acids as substrates for protein synthesis and carbohydrates are converted into energy through oxidative phosphorylation and citrate cycle.

### Hepatic metabolism in fetal and kid goats undergone maternal undernutrition

It has been well known that the liver is the central organ that plays multiple vital roles in hematopoiesis, detoxification and further nutritional metabolism, including protein, fat and carbohydrate metabolism (Grune et al., 1995). Previous studies have demonstrated that maternal undernutrition affected metabolism-related gene expression in sheep liver (Chadio et al., 2017) and changed hepatic transcriptome and plasma metabolic profile in lambs (Santos et al., 2018a). In the current study, maternal undernutrition upregulated the expression of genes associated with protein digestion and absorption in the fetal liver, which was in consistent with the results reported by *Santos et al. (2018a)*, who observed that early feed restriction during the suckling period downregulated gene expression involved in protein synthesis and protease inhibitors in lambs. These results suggested that feed restriction may lead to higher catabolism of proteins. Bile acids, the main component of bile, were synthesized in the liver, and secreted into duodenum to emulsify lipids and promoted the absorption of dietary liposoluble nutrients (Islam et al., 2011). The upregulated DEGs (*RUNDC3A*, *LOC102186539*, *LOC102173859*) enriched in bile secretion of our study suggested that feed restriction improve the digestion and absorption of dietary liposoluble nutrients to ensure energy metabolism in the liver of fetal goats. Meanwhile, carbohydrate digestion and absorption were promoted by feed restriction in fetal liver to provide energy for fetal goats, and steroid biosynthesis and thyroid hormone synthesis were all enhanced in fetal liver to ensure fetal growth and development. A pervious study has reported an increased capacity for protein synthesis in the liver of cattle after a feed restriction period (Keogh et al., 2016), which may because cattle underwent compensatory growth after realimentation. On the contrary, biosynthesis of amino acids in the kid goat livers of our study was restrained by feed restriction, though, kid goats might undergo compensatory growth after realimentation. These results suggested that maternal undernutrition may lead to programmed hepatic metabolism and the programming effect didn’t observe in a short time (fetal phase) but appeared after a long time (kid phase). *Guo et al. (2019)* observed that citrate cycle was significantly suppressed by severe feed restriction in sheep with lower level of glucose-6-phosphate, citrate and -ketoglutarate in the blood sample. Milk replacer restriction downregulated the expression of *isocitrate dehydrogenase 2* (*IDH2*) and *oxoglutarate dehydrogenase complex (OGDH)* which involved in citrate cycle of lambs (Santos et al., 2018b). These results were similar to the results of our study that feed restriction downregulated citrate cycle on transcriptome with lower expression of *succinate dehydrogenase complex subunit C* (*SDHC*) and *IDH1*. It indicated that the disorder of hepatic metabolism induced by maternal feed restriction appeared, which also had a programming effect on goats.

### Hepatic immune function in fetal and kid goats undergone maternal undernutrition

Except for metabolic function, liver plays a crucial role in immunity and hepatic lymphocyte that enriches in macrophages, natural killer and natural killer T cells to defense against pathogens and other antigens from gastrointestinal (Kubes & Jenne, 2018; Popescu et al., 2019; Stamataki & Swadling, 2020). In the current study, we obverted 4 and 6 immune-related DEGs in the liver of fetal and kid goats, respectively (Table 4). The results of KEGG pathway analyses for down-regulated DEGs mentioned above in the liver of fetal goats were involved in cytokine-cytokine receptor interaction and the NF-*κ*B signaling pathway. This finding is similar to the results of previous studies, which indicated that feed restriction (50% daily feed intake) from d 11 to 21 of gestation downregulated gene and protein expression of *TLR4* in rats (Alves-Rosa et al., 2002). Additionally, a 48-h feed deprivation upregulated the expression of *interferon-inducible cytokine IP-10* (*CXCL10*), *chemokine [C-C motif] receptor 9* (*CCR9*) and *C-C Motif Chemokine Ligand 2* (*CCL2*) in adipose tissue of lactating goats, which was different from our results that the expression of *MPL* and *CCL19* were downregulated by feed restriction (Faulconnier et al., 2011). These discrepancies suggested that malnutrition during gestation may limit protein synthesis and dysregulate *NF-κB* related innate immune response in the fetal liver. Another reason is that although goat received the same diet, variations among individual animals in the same group also existed that required to increase the number of animals in each group to avoid or reduce variation within the groups. Additionally, feed restriction decreased the expression of *C25H16orf96* in the liver of fetal goats, which was similar to the study of *Santos et al. (2018a)*, who showed that feed restriction led to a lower expression of *C1H21orf33* in the liver of lambs. However, immune-related pathways, including cytokine-cytokine receptor interaction and Toll-like receptor signaling pathway were downregulated by feed restriction in the liver of kid goats. These would explained that feed restriction might program metabolic processes in the kid goats, which lead to metabolic disorders, thereby activating the immune response. Additionally, gap junction, the channel link the cytoplasm of two cells that responsible for the exchange of ions, second messengers and small metabolites (especially glucose) between two cells (Meşe, Richard & White, 2007), was downregulated in the liver of kid goats, which may explained why citrate cycle was affected.

**Table 4:** Expression statistics (average FPKM) of the representative immune related DEGs in the of liver fetal and kid goats.

**Gene name**	**Gene expression**		**Fold change (log2)**	***P*value**	**FDR**
	**CON**	**RES**			
Fetal goats					
*MPL*	2.20	1.47	−0.584	0.001	0.068
*CCL19*	0.83	0.22	−1.932	<0.001	0.045
*PF4*	324.58	217.41	−0.578	<0.001	0.022
*C25H16orf96*	1.70	1.40	−0.283	0.002	0.079
Kid goats					
*GJD3*	0.45	0.16	−1.263	0.001	0.087
*TUBA1C*	53.32	44.01	−0.277	0.001	0.089
*C8B*	54.90	37.07	−0.567	<0.001	0.051
*C1S*	162.98	133.14	−0.292	<0.001	0.065
*CXCL10*	13.20	17.68	0.422	0.001	0.066
*IL1RN*	20.02	27.64	0.465	<0.001	0.020

As shown in the “thrifty phenotype” maternal undernutrition during gestation restricts fetal organs development and restrains fetal growth. It also leads to the reapportion of nutrients and energy to ensure the development of organs vital for fetal survival (brain, heart and lung) rather than other organs that less critical for survival (pancreas and kidney) which resulting in eventual maldevelopment of those organs in later life (Hales, Barker & Barber, 2001). Moreover, it is verified that maternal undernutrition during gestation increases the risk of developing “metabolic syndrome” in the juvenile stage in humans and animals, which is the so-called programming of metabolism (Gardner et al, 2005). Our findings indicated that down-regulated metabolic processes might be attributed to the “thrifty phenotype” or “metabolic syndrome” and maternal undernutrition may have a long-term impact on citrate cycle, carbon metabolism and biosynthesis of amino acids in kid goats after realimentation. On the contrary, maternal undernutrition only affected metabolic processes in fetal goats, and these metabolic phenotypes eliminated in kid goats (Zhou et al., 2019). The inconsistency between transcriptional and metabolic profiles implied that assessing the gene express levels more effective than plasma metabolites to reflect the long-term health induced by maternal nutrition when collecting samples from animals in early life. Moreover, we neglected the effect of gender on transcriptional profiles in livers of fetal and kid goats which may also be one reason for the difference between our study and our previous study.

## Conclusion

Hepatic transcriptome analysis showed that the significant changes at the level of transcription in the livers of nutrient restricted fetuses were related to metabolic and immune processes, which promoted protein digestion and absorption and suppressed immune process. However, nutritional restriction during mid-gestation mainly restrained the metabolic processes in kids’ livers. The results indicated that the effect of maternal undernutrition on the hepatic metabolism might not appear in fetal goat within a short time, however, this programmed effect on metabolic processes appeared in goat juveniles with ontogenetic growth and development. Transcriptome analysis gives us an insight into a molecular understanding of the nutritional restriction during mid-gestation on the hepatic metabolic function in fetal and kid goats that may provide the reference for further study on metabolism programming.

##  Supplemental Information

10.7717/peerj.10593/supp-1 Supplemental Information 1 Ingredients and nutrient composition of the diet for kids

10.7717/peerj.10593/supp-2 Supplemental Information 2 The primer sequences of genes related to hepatic metabolism and immunity in the liver of fetal and kid goats for qRT-PCR

10.7717/peerj.10593/supp-3 Supplemental Information 3 Sequencing results and percentage of reads mapped to reference genome of all groups

10.7717/peerj.10593/supp-4 Supplemental Information 4 List of expressed genes of each group in fetal and kid goats Expressed genes of FC group, FR group, KC group, and KR group.

10.7717/peerj.10593/supp-5 Supplemental Information 5 GO terms and KEGG pathways of top 3000 highly expressed genes among four groups

10.7717/peerj.10593/supp-6 Supplemental Information 6 Differentially expressed gene list of fetal and kid goats DEGs of fetal goats between KC and KR groups and DEGs of kid goats between KC and KR groups.

10.7717/peerj.10593/supp-7 Supplemental Information 7 GO enrichment analysis of differentially expressed genes (DEGs) between FC and FR GO enrichment analysis of upregulated and downregulated DEGs between FC and FR.

10.7717/peerj.10593/supp-8 Supplemental Information 8 Pathway analysis of differentially expressed genes (DEGs) between FC and FR Pathway analysis of upregulated and downregulated DEGs between FC and FR.

10.7717/peerj.10593/supp-9 Supplemental Information 9 GO enrichment analysis of differentially expressed genes (DEGs) between KC and KR GO enrichment analysis of upregulated and downregulated DEGs between KC and KR.

10.7717/peerj.10593/supp-10 Supplemental Information 10 Pathway analysis of differentially expressed genes (DEGs) between KC and KR Pathway analysis of upregulated and downregulated DEGs between KC and KR.

10.7717/peerj.10593/supp-11 Supplemental Information 11 FPKM value of hepatic metabolism-related genes. FPKM value of carbohydrate metabolism-related genes, lipid metabolism-related genes, and cellular energy metabolism-related genes in all groups.

## Additional Information and Declarations

### Field Study Permissions

The following information was supplied relating to field study approvals (i.e., approving body and any reference numbers): All goats used in this study belonged to a local farm (Mulei Black Goats Breeding Farm) in Liuyang, Changsha, China, and the owner (Mr. Yuanwei Yang) of the farm agreed to our experiment.

### Data Availability

The following information was supplied regarding data availability: Data are available at NCBI SRA: PRJNA595704 (accession number: SRP237643).
