# Supplementary material for: Transcriptome analysis reveals liver metabolism programming in kids from nutritional restricted goats during mid-gestation
Source: PeerJ. 2021 Jan 29;9:e10593. doi: 10.7717/peerj.10593 (PMC7849524; doi:10.7717/peerj.10593)
Supplement: Supplemental Information 1 [file peerj-09-10593-s001.docx]

| **Items** | **Content** |
| --- | --- |
| Ingredients (%) | |
| Fresh *Miscanthus* spp. | 20.00 |
| Maize | 36.00 |
| Soybean meal | 14.16 |
| Wheat bran | 14.40 |
| Whey powder | 6.40 |
| Fat powder | 6.40 |
| Calcium carbonate | 0.24 |
| Calcium bicarbonate | 0.80 |
| Sodium chloride | 0.40 |
| Premix ^a^ | 1.20 |
| Nutrient composition ^b^, % of DM basis | |
| Metabolic energy (MJ/kg) | 15.19 |
| Crude protein | 15.52 |
| Acid detergent fiber | 11.67 |
| Calcium | 0.76 |
| Phosphorus | 0.32 |

**Table S1 Ingredients and nutrient composition of the diet for kids**

^a^ Premix was provided per kilogram of total diet DM, and the composition was as follows: 95 000 IU of vitamin A, 17 500 IU of vitamin D, 18 000 IU of vitamin E, 119 g of MgSO_4_·H_2_O, 2.5 g of FeSO_4_·7H_2_O, 0.8 g of CuSO_4_·5H_2_O, 3 g of MnSO_4_·H_2_O, 5 g of ZnSO_4_·H_2_O, 10 mg of Na_2_SeO_3_, 40 mg of KI, 30 mg of CoCl_2_·6H_2_O.

^b^ Metabolic energy was calculated value.
