# Supplementary material for: Transcriptome analysis reveals liver metabolism programming in kids from nutritional restricted goats during mid-gestation
Source: PeerJ. 2021 Jan 29;9:e10593. doi: 10.7717/peerj.10593 (PMC7849524; doi:10.7717/peerj.10593)
Supplement: Supplemental Information 2 [file peerj-09-10593-s002.docx]

**Table S2 The primer sequences of genes related to metabolic and immune process in the liver for qRT-PCR.**

| Gene Name | Primer sequence (5’→3’) | Product size (bp) | Accession number |
| --- | --- | --- | --- |
| *Proproteinase E* | F: GTTGTCAATGGTGAGGATGCG | 94 | XM_018055348.1 |
|  | R: CGCCGCAGGTGTGATAAAAT |  |  |
| *COL16A1* | F: CGGGACACCCAGAACAATGA | 159 | XM_018057521.1 |
|  | R: GTGGGCCTTTGCTCCTCTT |  |  |
| *LBR* | F: TGTAAACAGAAGGAGCCCAGTC | 139 | XM_018060210.1 |
|  | R: TTCCAATCGGCAGTAAGT |  |  |
| *SLC2A5* | F: CTACTACTACGCAGACCAGATT | 131 | MH308027.1 |
|  | R: CCATAAGTTCCACCACGA |  |  |
| *CCL19* | F: CATTCCAGACAGCCTCAC | 157 | XM_005684095.3 |
|  | R: TCAAGGAGCAGGTAGCG |  |  |
| *PF4* | F: ATTCACCCCAGGCACATC | 97 | XM_013964718.2 |
|  | R: TTTTCCTCCCCGTCTTCA |  |  |
| *C25H16orf96* | F: GATGAGGGAACAACAGCA | 179 | XM_018040391.1 |
|  | R: AGTCCAACAACTCAGGGTC |  |  |
| *SDHC* | F: GGTATTGCCTTGAGTGC | 254 | XM_005677156.3 |
|  | R: GTAAGAACCAGGACAGC |  |  |
| *IDH1* | F: GACTGTCACCCGTCACTA | 179 | XM_018060230.1 |
|  | R: CAGCCTCAATGGTCTCAA |  |  |
| *TUBA1* | F: TTCGGAAACTGGCTGAC | 159 | XM_018047844.1 |
|  | R: GGGCTGGGTAAATGGA |  |  |
| *C8S* | F: ATCACGCCGTTGCCTAT | 296 | XM_005678354.3 |
|  | R: CTACAGTTCGCCTCATCT |  |  |
| *CXCL10* | F: TTCTGCCTTATCCTTCTG | 284 | NM_001285721.1 |
|  | R: TATGCCTCTTTCCGTGTT |  |  |
| *IL1RN* | F: CCAGAACAGGGAGCA | 143 | XM_005686683.3 |
|  | R: GCGTATTGGTGAGGC |  |  |
